# Feasibility and acceptability of living systematic reviews: results from a mixed-methods evaluation

**DOI:** 10.1186/s13643-019-1248-5

**Published:** 2019-12-14

**Authors:** Tanya Millard, Anneliese Synnot, Julian Elliott, Sally Green, Steve McDonald, Tari Turner

**Affiliations:** Cochrane Australia, School of Public Health and Preventative Medicain, Monash University, Melbourne, Australia

**Keywords:** Living systematic review, Systematic review, Methods

## Abstract

**Background:**

Living systematic reviews (LSRs) offer an approach to keeping high-quality evidence synthesis continually up to date, so the most recent, relevant and reliable evidence can be used to inform policy and practice, resulting in improved quality of care and patient health outcomes. However, they require modifications to authoring and editorial processes and pose technical and publishing challenges. Several teams within Cochrane and the international Living Evidence Network have been piloting living systematic reviews.

**Methods:**

We conducted a mixed-methods evaluation with participants involved in six LSRs (three Cochrane and three non-Cochrane). Up to three semi-structured interviews were conducted with 27 participants involved with one or more of the pilot LSRs. Interviews explored participants’ experiences contributing to the LSR, barriers and facilitators to their conduct and opportunities for future development. Pilot team members also completed monthly surveys capturing time for key tasks and the number of citations screened for each review.

**Results:**

Across the pilot LSRs, search frequency was monthly to three-monthly, with some using tools such as machine learning and Cochrane Crowd to screen searches. Varied approaches were used to communicate updates to readers. The number of citations screened varied widely between the reviews, from three to 300 citations per month. The amount of time spent per month by the author team on each review also varied from 5 min to 32 h. Participants were enthusiastic to be involved in the LSR pilot. They highlighted the importance of a motivated and well-organised team; the value of technology enablers to improve workflow efficiencies; the need to establish reliable and efficient processes to sustain living reviews; and the potential for saving time and effort in the long run. Participants highlighted challenges with the current publication processes, managing ongoing workload and the lack of resources to support LSRs in the long term.

**Conclusions:**

Findings to date support feasibility and acceptability of LSR production. There are challenges that need to be addressed for living systematic reviews to be sustainable and have maximum value. The findings from this study will be used in discussions with the Cochrane community, key decision makers and people more broadly concerned with LSRs to identify and develop priorities for scale-up.

## Background

Timely use of reliable research evidence is required for optimal health care; however, there is a persistent gap between research findings and healthcare practice. As a consequence, many patients are continuing to receive sub-optimal care [1]. While systematic reviews and meta analyses are profoundly beneficial in addressing this gap, there are significant limitations restricting their benefit including the long production time; the fact that many reviews are out of date on publication; that conducting a review update can be similar in workload to starting the review again; and that only a minority of reviews are updated within two years of publication [2–5]. This inability to maintain currency results in significant inaccuracy.

Living systematic reviews (LSRs) offer an approach to keeping high-quality evidence synthesis continually up to date, so the most recent, relevant and reliable evidence can be used to inform policy and practice, resulting in improved quality of care and patient health outcomes. Living systematic reviews are systematic reviews that are continually updated, incorporating new, relevant data as it becomes available [6]. LSRs can be enabled by new technologies, such as use machine learning and automation, and crowd sourcing or citizen science initiatives to increase efficiency in areas such as screening of titles and abstracts, substantially reducing workload for review teams [7]. By retaining methodological rigour while staying on top of new evidence, LSRs break the historical trade-off between review quality and currency. Living systematic reviews offer a new approach to review updating, and present exciting new opportunities for similar initiatives, such as living guidelines and living recommendations.

Ensuring these high-quality evidence syntheses are continually up to date requires some modifications to existing authoring and editorial processes, and poses a number of technical and publishing challenges. In 2016, the Living Evidence Network was initiated to bring together those involved in Cochrane’s first pilot LSRs. The Network now consists of more than 200 people both within and outside Cochrane. Several Cochrane teams and others in the Living Evidence Network have been piloting living systematic reviews. There are currently five LSRs published in the Cochrane Library and the Network has published guidance for conduct of LSRs. We aimed to explore the experiences of those conducting pilot LSRs and to assess the feasibility and acceptability of this new approach in order to refine future LSR production models.

## Methods

This study aimed to assess the feasibility and acceptability of LSRs and explored how people are currently conducting LSRs, the facilitators and challenges, opportunities for improvement and considerations for scale up. We used a mixed methods approach that included semi-structured interviews and online surveys with key members involved with pilot LSRs. The study was approved by the Monash University Human Research Ethics Committee.

Six LSR teams were invited to participate in this evaluation study. Purposive sampling was used to recruit review teams who were known to be conducting or proposing to conduct an LSR at the commencement of the pilot period. Online surveys and semi-structured qualitative interviews with key members involved with the pilots explored their experiences with LSRs. The pilots covered several types of systematic review, including intervention effectiveness, cross-sectional and guideline adherence.

The Cochrane LSRs included:

- Anti-coagulation in people with cancer [8];
- Fruit and vegetable consumption in children [9]; and
- Delayed antibiotics for respiratory infection [10].

Non-Cochrane LSRs included:

- Zika virus and adverse neurological outcomes (F1000Research and PLOS Medicine) [11];
- Adherence to guidelines in traumatic brain injury (Journal of Neurotrauma) [12]; and
- Epidemiology of traumatic brain injury (Journal of Neurotrauma) [13].

### Interviews

Up to three semi-structured interviews were conducted with each of the key team members involved with the LSR pilots between September 2017 and August 2018. Twenty-seven participants were interviewed including lead/senior authors (*n* = 8), information specialists (*n* = 3), those responsible for managing the review production and publication process (managing editors, *n* = 3) and coordinating the review production for clinical areas within Cochrane (Coordinating Editors, *n* = 3), peer reviewers (*n* = 2), other editorial team members involved in the LSR pilots (*n* = 6), the project lead for Cochrane Crowd (*n* = 1) and the coordinator of the pilot living systematic reviews (*n* = 1).

The interviews explored participants’ experiences of conducting/contributing to an LSR and the barriers, facilitators, challenges and advantages of LSR processes. Interview questions were loosely based on a predetermined interview schedule, with questions varied to suit the interviewee’s roles and experience. Participants were asked about their role and contributions to the LSR they are involved in, key learning’s, the processes they follow, the aspects of the process which work well and those which are more challenging. They were also asked about opportunities for improvement. The interview schedule was developed by TM in consultation with the other authors. Interviews were conducted via online meeting software or by phone and digitally recorded. Interviews were conducted by TM, TT or AS all of whom worked on the LSR pilot program within Cochrane, none of whom have authoritative roles within Cochrane and all of whom have extensive experience in qualitative interviewing. Interviews had an average duration of approximately 30 min. Interview recordings were reviewed for familiarisation and key passages transcribed verbatim. The authors felt that partial transcription was suitable for the type of analysis needed for the evaluation. Additional relevant information from field notes was added to the transcriptions.

### Surveys

Surveys were distributed each month from October 2017 to July 2018, commencing after each pilot team had published their baseline LSR. Quantitative and qualitative data were collected using an online survey tool (Qualtrix). The surveys were tailored to each group of respondents (i.e. author, editor or information specialist) and captured the time spent on monthly LSR tasks (all), the number of citations screened per month (authors/information specialists) and key learnings and reflections throughout the pilot period (all). A minimum of one participant from each team was asked to complete the online monthly surveys. Respondents were the senior/lead authors (six) from each LSR author team. For the three Cochrane LSR teams, the respondents also included the information specialists and managing editors.

### Data analysis

NVIVO 12 was used to analyse the data and extract quotes. The data was thematically analysed using both an inductive and deductive approach. Transcripts were read and re-read for familiarisation. An initial set of codes were developed by TM, and verified by AS and TT. Some codes were identified as a priori, using the interview schedule while others emerged inductively from the data. Coded extracts were collated into emerging themes which were reviewed and refined through discussion between the study team. TM undertook the primary data analysis. TT and AS reviewed and collaborated on the conceptual development and refining of themes. A draft report of the analysis was provided to the Living Evidence Network (which included interview respondents) for feedback. The data from the open-ended questions in the surveys were combined with the interview data due to the similarity of themes. Quantitative data obtained from the surveys were analysed using simple descriptive statistics.

## Results

### Characteristics of living systematic reviews

As previously discussed, the reviews were a mix of Cochrane and non-Cochrane reviews. Across the pilot reviews, search frequency was monthly to three-monthly, with some LSR teams using machine learning and Cochrane Crowd to screen records. Between two and four authors were involved with ongoing maintenance of the review. Each of the Cochrane review teams included information specialists to develop and run the monthly searches and, along with the editors, provide ongoing LSR methods support. The non-Cochrane review teams were responsible for running their own searches. Some funding for personnel was received by five of the reviews. Varied approaches were used to communicate updates to readers daily, monthly or 3–6 monthly. Variations in editorial and peer review procedures and the processes and triggers for the integration of new evidence existed across the reviews (Table 1).

**Table 1:** Key characteristics of the living systematic review pilot teams, processes and publication models

Review topic^a^	Anti-coagulation in people with cancer (3 related LSRs)	Fruit and vegetable consumption in children	Delayed antibiotics for respiratory infections	Zika virus and adverse neurological outcomes	Adherence to guidelines in traumatic brain injury	Epidemiology of traumatic brain injury
No. of authors maintaining LSR^b^	4	2	2	4	2	4
Search or other support	Information specialist to develop and run searches; ongoing LSR methods expert support			Librarian to develop searches only	Information specialist to develop and review searches only; ongoing LSR methods expert support	
Direct funding for personnel	Yes (Part-time RA for authors)	Yes (Part-time RA for authors, stipend for editorial group)	No	Yes (Three funded positions [various roles] for authors)	Yes (Authors funded as part of broader work program, plus specific part-time LSR methods expert)	
Journal/Editorial Group; Publisher	Cochrane Gynaecological, Neuro-Oncology and Orphan Cancers; Cochrane	Cochrane Heart; Cochrane	Cochrane Acute Respiratory Infections; Cochrane	F1000Research; F1000	Journal of Neurotrauma; Mary Ann Liebert, Inc.	
Search frequency	Monthly^c^			Daily or monthly^d^	Three-monthly^e^	
Technological enablers^f^	Machine classifier and crowd-sourcing to identify RCTs^g^		Nil	Automation and machine learning algorithms to identify RCTs, with some data output automation^h^	Nil	
Communicating review status to reader	Monthly statement to reader about review status (i.e. search date, new studies found, update plans) published in the ‘What’s New’ section of the review, via an article amendment			Daily updates (search date, new studies found) via study website^i^	3–6 monthly updates, describing results of new evidence found, available as supplementary material in online version of article	
Editorial and peer review of status updates	No formal editorial review; no peer review			No formal editorial review; no peer review	Editorial review and copy-editing; no peer review	
Process for integration of new evidence (citation/DOI status)	Full re-publication of review, with new citation and DOI^j^			New version of the review published, with linked citation and DOI (intended)^k^	Full re-publication of review, or short commentary article, with a new citation and DOI (intended)^k^	
Editorial and peer review of new versions/publications	Standard editorial and peer review processes apply (may qualify for ‘selective’ peer review per Cochrane policy); same peer reviewers approached			Standard editorial and peer review processes apply; same peer reviewers approached (intended)^k^	Not confirmed (likely standard editorial and peer review processes apply) (intended)^k^	
Trigger for integration of new evidence	When new evidence identified that changes review conclusions (intended)^k^	Every 4 months (irrespective of impact of new evidence)	When new evidence identified (irrespective of its impact) (intended)^k^	Every 6 months (irrespective of impact of new evidence) (intended)^k^	When new evidence identified that changes review conclusions, but no more frequently than yearly (intended)^k^	

*DOI* digital object identifier, *LSR* living systematic review, *RA* research assistant, *RCT* randomised controlled trial

^a^Each team produced one LSR unless otherwise stated

^b^Meaning authors who contributed to the ongoing review tasks associated with maintaining the LSR (this may or may not have included the entire author team who contributed to the ‘baseline’ review)

^c^Electronic databases ± clinical trials registries all searched monthly, with remaining non-database sources, such as journal hand searching, websites and conference proceedings searched every 6 months

^d^Daily searches for PubMed, Embase and LILACS databases, with monthly searches for all other sources

^e^All sources (including non-database sources) searched at this frequency

^f^‘Technological enablers’ refers to both computer technology and more efficient models of human contribution to increase the efficiency and sustainability of the systematic review enterprise (adapted from Thomas 2017 J Clin Epi 91:31-37)

^g^Cochrane Crowd is a citizen science platform used to screen titles and abstracts to identify relevant citations (RCT’s) and exclude irrelevant citations. Machine classifiers exclude irrelevant citations automatically by using algorithms to predict how likely a new citation is to be describing a RCT

^h^Searches in some databases (PubMed, Embase and LILACS) are automated. De-duplication of citations is automated. Machine learning algorithm suggests a decision for inclusion based on title and abstract. All existing predefined tables and figures can be updated by running a script that re-renders these tables and figures

^i^Study website is the Zika Open Access Project, available at: https://zika.ispm.unibe.ch/home

^j^This process was implemented in one Cochrane Review only (Fruit and vegetable consumption in children). It was the intended process to be used in the remaining Cochrane Reviews but they did not reach the trigger for integration of new evidence, so their reviews were not re-published during the pilot period

^k’^Intended’ refers to the fact that this was the agreed process and/or trigger for integrating new evidence but that it was not undertaken during the pilot period

### Review progress and workload

Between seven and 101 studies were included in the baseline publications of the LSR’s and five to 96 new studies were identified during the pilot period. Between zero and ten status updates were provided after the baseline review and only one review was formally re-published (Table 2).

**Table 2:** Review progress and workload implications during the pilot period

Review topic^a^	Anti-coagulation in people with cancer (3 related LSRs)^b^	Fruit and vegetable consumption in children	Delayed antibiotics for respiratory infections	Zika virus and adverse neurological outcomes	Adherence to guidelines in traumatic brain injury	Epidemiology of traumatic brain injury
Date ‘baseline’ review published	LSR 1^c^: September 2017 LSR 2^d^: December 2017 LSR 3^e^: June 2018	September 2017	September 2017	February 2018	October 2015	November 2015
No. of studies in ‘baseline’ publication	LSR 1^c^: 19 LSR 2^d^: 7 LSR 3^e^: 16	50	11	101	22	66
No. of new studies found since ‘baseline’ publication^f^	5^g^	13	0	96	14	18
No. of status updates since baseline review	Monthly updates LSR 1^c^: 10 LSR 2^d^: 7 LSR 3^e^: 1	10	10	0	4	3
No. of times review re-published	0^7^	2 (January and May 2018)	0	0	0	0

*No*. number

^a^Each team produced one LSR unless otherwise stated

^b^These three LSRs were part of a suite of LSRs that used a single search

^c^Refers to the first in the suite of LSRs published: Parenteral anticoagulation in ambulatory patients with cancer.

^d^Refers to the second in the suite of LSRs published: Oral anticoagulation in people with cancer who have no therapeutic or prophylactic indication for anticoagulation.

^e^Refers to the third in the suite of LSRs published: Anticoagulation for the long-term treatment of venous thromboembolism in people with cancer.

^f^Includes new (not ongoing) studies that were screened and found to meet the inclusion criteria for the review until the end of the pilot period

^g^Data presented for all three reviews (as appropriate, given they transitioned into living mode at different times during the pilot period) given the same search fed into all reviews and they were managed by the same author and editorial teams

In looking at the workload during the pilot period, the number of citations screened varied widely between the reviews, from three to 300 citations per month. The amount of time spent per month by the author team on each review also, predictably, varied widely, from 5 min to 32 h, depending on both the screening workload, and whether the review was being updated and republished that month. There was no clear pattern in how this time was spent on different tasks in the review process, and interpreting the data was complicated by searches that covered multiple reviews, different search frequencies and varying approaches to workflow for screening and inclusion.

For Cochrane LSRs, the time spent per month by Managing Editors varied over a smaller range, from 0 min to 3.5 h, with larger workload associated with LSRs that were being republished. Similarly, the time spent by Information Specialists varied from 30 min to 6 h per month.

The results of the qualitative analysis are presented below. Overarching themes that emerged include motivations for undertaking living systematic reviews, initial expectations, overall experience, benefits, enables, efficiencies, challenges and opportunities for improvement. Team roles have been presented in place of pseudonyms for direct quotes.

### Motivations for undertaking living systematic reviews

Participants described many reasons for undertaking an LSR. All identified the novel appeal of LSRs and the notion of reduced workload and increased efficiency as a source of motivation. Many were interested in the methods for systematic reviews and keen to explore how the process could be conducted more efficiently. The potential for time-saving compared to standard approaches to updating SRs was appealing.

For me, LSRs are an interesting, novel concept. I am intrigued about the notion that it might reduce workloads compared to standard reviews. (Information specialist)

For all participants, the overwhelming appeal of undertaking an LSR was to increase the reliability of reviews—ensuring they remain current and based on the most recent research which can then better inform decision making, policy and practice.

Keeping the review up to date and relevant for people using it is very appealing. (Cochrane author)

Enthusiasm (for LSRs) has been driven by everyone’s commitment to evidence based medicine. There is no solution to systematic reviews going out of date. The volume of evidence, potential of LSRs and overwhelming need for this to happen. (LSR coordinator)

Participants frequently mentioned the high profile of LSRs as a motivating factor. Publicity, profile and recognition for authors along with the increased number of research outputs for the author team were also considered motivating factors in undertaking an LSR. Participants described their excitement at being involved in piloting LSRs and having the opportunity to contribute to process refinement.

It is a very interesting area and a great learning opportunity. It is also an opportunity to influence how they are being done. (Cochrane author)

### Initial expectations

Many participants began the process of conducting/contributing to an LSR with a sense of uncertainty, particularly around the impact on their workload. Authors were largely hopeful, yet sceptical, that LSRs may prove more time efficient than traditional review updates. They identified that the increased frequency of tasks would likely increase their workload, however, believed that this increased frequency could potentially make LSRs more manageable in the long run, compared with the arduous and highly time-intensive one-off task of updating a traditional systematic review. Participants expected that the LSR process would be a learning curve and present some ‘teething issues’ as the process evolves and is streamlined. A few participants described apprehension about engaging with the new technologies.

The feeling at the beginning was kind of half daunting and slightly overwhelming but also feeling very supported by the strategic group who was running the project (Cochrane author).

I’m a bit worried going forward. I have some vague notions about the process but I don’t feel certain about how it’s going to play out or the impact workwise (Information specialist).

The authors will have smaller sets of results to screen rather than having a huge overwhelming set of results and finding time once a year or 2 to work through rather than topping it up incrementally. It might actually be a bit more efficient for them. For me, it is a little more time consuming because I have to do a search each month but because it will be a smaller number of results, hopefully it will be quicker in terms of importing and exporting etc. (Information specialist).

### Reality—overall experience

Overall, participants were highly enthusiastic and largely positive about their experiences in the pilot LSR. They reported feeling well supported throughout the process and largely felt that the process itself was ‘quickly demystified’ and ‘not as difficult as originally predicted’. They described their contribution to an LSR as a fantastic learning opportunity and an interesting academic experience, many discussing opportunities for new collaborations that arose as a direct result of their involvement.

I felt very involved in contributing to the paperwork and the practical logistics of how an LSR should be done and what it would look like. It’s been very exciting. (Information specialist)

Everyone has been highly enthusiastic, willing to experiment and prioritise. They have been overwhelmingly responsive and proactive. (LSR coordinator)

Participants noted that the response from the community to LSRs has been overwhelmingly positive, with high levels of interest and excitement, and a sense of novelty about living systematic reviews.

Overwhelmingly, the main concerns expressed by participants at the end of the pilot period surrounded managing the ongoing workload and refining publication methods/processes. In terms of impacts of the LSR workflow on the separate stages of systematic reviews, the main area highlighted was the search stage.

### Benefits

When talking about benefits of LSRs, common themes included the rapid identification and translation of research evidence; the benefits to Cochrane directly; the continual, live process; and the improved accountability and commitment to the review.

Participants highlighted the appeal of up to date evidence. They discussed LSRs as resulting in the rapid identification of new evidence and the ability of this evidence to inform future decision-making, guideline development and clinical practice. The live, dynamic nature was seen as a significant benefit.

The evidence base for our topic was very small… There is now a large amount of information to inform practice, most of which has been integrated. (Cochrane author)

It has been very interesting to see the evidence base change over a short period of time. (Cochrane author)

In the LSR where no new trials were identified during the pilot period, the authors found the process to be very low maintenance. Although no new studies were identified, they felt it was good to be able to say with confidence that the review was current.

Living systematic review processes ensure that the author team is up to date on the newest evidence, integrating the evidence into their reviews, and tweaking the conclusions and citations constantly. For many, this continual, live process resulted in author teams feeling ‘on top of the curve’. The authors get to see the picture developing over time rather than a large amount of evidence delivered and processed at the end of an update.

There are typical shifting milestones, this is more steady and predictable. You are forced to keep up to date despite other tasks. (Cochrane author)

### Enablers/facilitators

In discussing the enablers or facilitators in the living systematic review processes, participants identified the importance of team enthusiasm and commitment; and input from Cochrane’s living systematic review team and the Living Evidence Network.

The LSR process heavily relies on the ongoing commitment of the author team, editorial team and publishing team, and their immediate capacity and skills. The benefit of having one team responsible for an LSR, and this responsibility being documented with very strict timelines, was believed to result in improved responsibility/accountability and commitment to the review. The strict timeline meant that people needed to prioritise the review over other tasks and adhere to the specified timeframes. While some considered this a benefit, it was also seen as a significant challenge (and further described in challenges section).

High levels of organisation, motivation and team commitment were identified as requirements for LSRs, needing to ensure that the work process is clear so everyone knows exactly what they have to do, and the timeframe for completion.

It is a complex moving process which requires strong attention to detail, a high level of communication and a coordinated management approach. (Cochrane author)

Participants described the enthusiasm and support they received from everyone involved (from authors to the editorial team and publishing members) as being key facilitators and essential to the success of the LSR. Team responsiveness and communication were seen as vital to keep on track and adhere to tight deadlines. The high level of enthusiasm and commitment facilitated the constant communication between the team members and with the editorial teams which was seen to be vital to the conduct of LSRs and key to their efficiency.

I felt very supported by the strategic group who was running the project. There was always someone to ask a question and someone with an answer and if not, an answer was quickly forthcoming. (Cochrane author)

The input from Cochrane’s living systematic review team was repeatedly highlighted as a significant enabler to the success of the Cochrane LSRs. Participants described feeling supported through the process and the benefits in being involved in discussions with a wider team concerned with LSRs. The role of the living systematic review coordinator in linking everyone, clarifying processes and resolving issues was highly valued and many participants expressed the need for this support to continue in the future.

The living systematic review team were constantly providing support, encouragement, pushing, motivating and keeping everyone moving. My question is to what extent that will be able to be there in the future? (Cochrane author)

Participants also emphasised the role of the Living Evidence Network in providing additional support, learning opportunities and opportunities to work with people with different skills in LSRs and thus, facilitating knowledge exchange and professional development. The large number of people involved in the Network was seen to give access to increased expertise and increase overall benefit to all of the pilot LSR teams.

The involvement of experts built the legitimacy of LSR and increased the feasibility of the model. (Information specialist)

It’s fantastic to feel part of a wider group. The emails, suggest fest, etc. were great. We felt very well supported. (Cochrane author)

It has been a good experience working with people with different skills in LSRs and the opportunity for knowledge exchange. (Information specialist)

### Efficiencies

In describing factors which increased efficiency, the most common themes included the repetitive nature of the process, team responsiveness, automation in searching and having an information specialist.

The potential for overall time and effort saving for researchers was flagged. Participants expressed that as the pilot progressed, the whole process became more streamlined, that the repetitive nature and increased familiarity with the processes increased their efficiency. Continually being aware and across the review was seen as a key factor contributing to the efficiency of LSRs.

Having your head in a body of literature every month can only mean increased efficiency (Cochrane author).

Constant communication between the teams themselves and with the managing editors was mentioned as vital to the conduct of LSRs and key to their efficiency. The high level of support and responsiveness provided by individual team members and the Cochrane LSR coordinator meant that immediate questions were answered thus preventing delays in the process.

We have worked with a really good author team, very active and engaged and that has been the key to success (Information specialist).

Efficient team is key to feasibility. Need speed of communication to make crucial decisions and progress. Big communication gaps cannot occur (Information specialist).

Consistent correspondence with the author team has made the process feel more connected, more alive (Cochrane managing editor).

Despite initial challenges and some remaining areas for improvement, the Cochrane LSR teams identified the search phase as the easiest or most efficient component of the LSR process, particularly those aspects that are automated or technologically assisted. Information specialists described the benefit of saving searches in databases and setting up automatic alerts to receive the monthly searches. New and supporting technologies, including Cochrane Crowd, Covidence and Cochrane Register of Studies (CRS), were all seen as increasing efficiency of the living systematic reviews. The ongoing challenges with the integration and use of these technologies were discussed along with suggestions for improvements (presented below).

We need to develop skills and faith in the use of these technologies (including CRS Web). This may present further time saving. (Cochrane author)

I feel that we have used the machine aspects well and in addition to Cochrane Crowd to reduce both the burden and magnitude of time spent on screening records. (Cochrane author)

A major strength is that I can run and collate the results myself and check them against previous results sent to ensure the authors workload is kept as small as possible. (Information specialist)

The involvement of an information specialist, as seen in the Cochrane groups, may be key to the search efficiency as the non-Cochrane groups all identified ongoing issues with screening or a large burden on authors as a result of screening tasks (see explanation in ‘Challenges’ section).

It is a lot of ongoing work. I’m constantly juggling my time, managing a high number of citations and a high number of irrelevant hits. (non-Cochrane author).

We need someone to double screen the citations. Searching the databases takes a lot of time (non-Cochrane author).

The major enabler is having someone run the search and send me a ready made EndNote library (Cochrane author).

### Challenges

In discussing their experiences, several challenges of the living systematic review process were highlighted. Themes included the ongoing workload, issues with search and screening and editorial/publication issues.

#### The ongoing workload

The ongoing workload was perceived as requiring a large investment and ensuring immediate availability of capacity was considered to be a significant and sustained challenge, particularly for members of the author team. Participants discussed a variety of tasks as contributing to the workload including tracking ongoing studies, locating full text articles, chasing trial authors for data, issues with screening and data management, updating PRISMA and results tables and the publication process. Many of the team members described feeling stressed and, at times, frustrated with the challenge of keeping up. They questioned author capacity and motivation to perform all of these tasks in the long term, particularly in the absence of additional funding.

Updating the manuscript every three to four months requires a large capacity over a short period of time. In the long term this may become an increasing challenge especially without funding to support sustainability. (Cochrane author)

Author teams need to have the review in the front of their consciousness all the time*—*this won’t work for all author groups. For example, [I suggested to one of the groups] they might like to pilot an LSR *‘*it’ll be fun!,*’* but they were put off by the idea of having to keep the review in their consciousness… It was unexpected that author teams were lukewarm to the idea. (Cochrane managing editor).

We are concerned about the human capacity to maintain the review(s). We need to do continual updates as opposed to updating every three years. The availability of people familiar with the review and the process is important. (Cochrane author)

Without extra resources, this level of engagement and investment is probably unsustainable. (Cochrane author)

Many participants described the process as feeling very rigid and not providing a lot of leeway. Authors described the constantly revolving process as time consuming and, at times, cumbersome.

The process required multicomponent, ongoing tasks. At times we were conducting our monthly search, plus addressing comments, plus integrating new findings all at the same time. This was way more complicated than expected. (Cochrane author)

The complexity of having a manuscript updated and continuing with monthly screening is a challenge. Contacting authors can result in delays. There are multiple concurrent aspects of the review updating at once*—*this is tricky but required. (Cochrane author)

There are many documents to update and screen on a monthly basis. Even if eligible studies are not identified the PRISMA still needs to be updated. (Cochrane author)

It is a job that never ends. It is interesting but I have other deliverables also. (Cochrane author)

There is not a lot of flexibility in the approach*—*What happens if leave is taken by key member? (Information specialist)

Largely, workload issues experienced by participants outside of the author teams resulted from publication frequency and then need for methodological guidance rather than the burden of work.

#### Editorial team challenges

Cochrane Managing Editors described having a very experienced author team as making the process efficient. They had the LSR Guidance manual to follow and largely found limited changes required to their standard editorial processes. They described the couple of weeks leading into the first publication as intensive as it was the first time they were publishing an LSR and the small changes to the review required multiple checks to ensure they were correct.

Initially, the publication timing, keeping up with the speed of everything caused a lot of frustration within the team. Now there is an increased workload but it is not necessarily more cumbersome. The nature of the monthly work is different and feels more manageable. (Cochrane managing editor)

Securing peer reviewers was identified as an ongoing challenge and editors emphasised the need to set up peer review in advance in order to prevent delays. The peer review process was reliant on the reviewers adhering to the strict timelines for the LSR. Overall, the editorial process was largely considered ‘similar to other publications but expedited all round’. Managing Editors reiterated the need for additional resources (namely funding) to support the sustainability of LSR from an editorial perspective.

The turnaround time is also difficult (it’s hard enough when you get 2-4 weeks with other manuscripts) and it is already more time-consuming because it is a Cochrane Review. (Peer reviewer)

I like the idea of repeat peer reviewing the same manuscript. Over time, you would become more familiar with the topic and you would get to see how if the peer review you are providing is helpful and how that might be changing the manuscript. So it would be nice to get that feedback. Sometimes being a peer reviewer feels mean for the sake of being mean, and this way it’s like you are more associated with the LSR and taking some responsibility for it. (Peer reviewer)

From a copy-editing perspective, challenges included substantially increased workload and insufficient resources to support this. The need for advanced communication about the arrival of a review for copy-editing within the tight timeframes designated by the LSR process was emphasised. Dangers of inconsistency were highlighted, with the potential for copy-editors to rush the process to adhere to deadlines. As with other editorial tasks, the time required for copy-editing was substantially reduced for each subsequent version.

#### Challenges with search and screening

For information specialists, the need to refine the process to ensure efficiency was paramount. They described the monthly search surveillance as initially intensive to set up, but once in place, was viewed as an ‘efficient, reliable, predictable process’. However, the increased frequency of searching added considerably to their workload.

The search for a traditional systematic review update takes between 1 to 5 days and then you are done for two years. With LSRs you receive constant emails with new citations over the month (which you need to organise) and then you need a morning of work to process the citations and pull them all together for the authors (Information specialist).

It feels very rigid. There is the constant receipt of monthly alerts and the need to plan for them. There is not a lot of leeway (Information specialist).

Information specialists described significant challenges in learning how best to manage and organise the constant flow of new citations. Teams used different approaches to retrieve and screen the most recent evidence, with some using machine learning (RCT classifier) and citizen science (Cochrane Crowd) to reduce the screening burden. Technical issues, such as managing auto-alerts and discrepancies in Crowd assessments, were largely resolved and not considered ongoing issues once the technology was integrated within regular workflows. Some information specialists expressed the need to develop their skills in using these technologies to establish trust and overcome concerns with their reliability.

Participants highlighted the potential of these technologies to result in further time saving. For some, the technologies they initially used did not meet all of their needs or work out as planned, resulting in the teams reverting to manual completion of the tasks. The information specialist in one of the pilot groups decided against using technology to assist screening due to the very small monthly yield of records. They believed that the tasks were not big enough for automation to make a difference and would have resulted in double handling.

For the non-Cochrane teams, search was the responsibility of the author team and was a significant contributor to the workload. One author was keen to use the new technology enablers to reduce the screening but since these focus on identifying randomised trials, there are limits to their use in reviews that include non-randomised designs. The authors of non-Cochrane LSR pilots reported feeling that they currently do not have the resources, technology or tools to manage the frequent searches and minimise the workload. The need for further refinement of existing technologies and the development on new innovative tools was emphasised.

We had issues with hits via automatic database searches. Screening automation causing issues and a large number of citations to screen (Non-Cochrane author).

We attempted to alleviate (the high workload) with technology however it remained a challenge. Diverse data (including more than RCTs) meant that the search algorithms did not work so well (Non-Cochrane author).

#### Publication **i**ssues

Cochrane LSR teams identified substantial issues with the publication process. Delays with implementing changes to the publication interface meant that it was not obvious which reviews were LSRs, when they had been updated and what this involved. Members of the author teams all described their disappointment and frustration with this delay. The need to clearly highlight within the review (and updates) what is new, what has been found and what has been included was emphasised.

Frustration was also expressed with the current process of republishing reviews triggering a new DOI, negatively affecting citation counts and impact factor. One of the pilot teams, which identified a high number of new studies, published frequent updates. Several versions of the review were published in a small amount of time with ‘not much difference between versions … this feels like a lot of work for a diminishing return’.

It was really difficult to go into the workflow in Archie and work out what happened when, and where the review is currently up to because of all the amendments being published every month, in addition to the other tasks for re-publication of full updates (Editorial team member).

Issues with the publication processes were also flagged by the non-Cochrane participants. They faced delays due to needing institutional clearance and ‘classical publication issues’ including copyright issues with authors which were largely outside of their control. As a consequence of these delays, one LSR pilot team reduced their update frequency from monthly to every 3 months.

A major barrier in speed for the sexual transmission review was the time spent at clearance at different US institutions (Non-Cochrane author).

By the time the update gets to the reviewers, it is already dated (Non-Cochrane author).

#### A lack of clarity around authorship

Several team members discussed authorship issues as a complexity in LSR production. They described the original reviews as having large authorship teams while the smaller, more nuanced changes to living review required a much smaller team. The opportunity for contribution is further restricted by the speed of the updates. This led the author teams to question when people should come off the author list and requesting more guidance around this issue.

LSRs require a smaller team due to low the volume of high frequency, rigid work (Cochrane author).

### Opportunities for improvement and scale up

In discussing opportunities for improvement and scale-up, key themes included the need for additional guidance, changes to current publishing processes, the refinement and development of technology to reduce workload, the need for resources and support and encouraging/facilitating knowledge translation.

#### Guidance

Many of the challenges with the current LSR processes raised by participants indicate the need for further clarity about the responsibilities of different contributors to LSRs and the LSR methods and processes. Participants suggested the need for guidance on:

- Eligibility/criteria for reviews to become living systematic reviews
- When and how LSRs cease to be living, and what happens then
- Evolving authorship
- Automation/tech tools available
- Submission processes and editorial policies
- Peer review—how to approach reviewing an LSR
- Methods for publication

Feasibility may be limited by others’ fear of the unknown. People seem sceptical about time consuming tasks. We need to demystify the process and continue to provide support (Cochrane senior editor).

Overwhelmingly, the most frequently mentioned consideration with regards to scale-up was the need for additional guidance about the appropriateness/prioritisation/selection of a review to become an LSR. Participants emphasised the need to select/prioritise reviews based on feasibility and impact and suggested ‘cherry picking’ topic areas for LSRs. One participant suggested concentrating on reviews that are listed as a priority for Cochrane review groups. The potential for ‘targeted living reviews’ was also mentioned. Largely, participants indicated that LSRs should focus on areas that have a large number of emerging trials or a constantly changing evidence base.

We need very specific criteria about appropriateness of a review to become an LSR. They are appropriate when the evidence base is uncertain and health decisions based on the findings have important outcomes (Cochrane author).

We need to prioritise reviews based on feasibility and impact. If the field is moving quickly and there are highly engaged authors in well supported groups... (Cochrane author).

We need to prioritise the right questions, and not be led by researcher enthusiasm. Perhaps we could tie to Network priorities (Cochrane senior editor).

Look at the topics—seek clinical experts’ advice on need for living mode (Information specialist).

Several participants highlighted LSRs as being particularly useful in responding to disease outbreak and suggested that guidance or a protocol for conducting an LSR in this circumstance could be beneficial.

They are highly applicable for disease outbreak. The evidence and questions need to be highly relevant to the current context. Tease out the bits which are specific to this outbreak and target the review accordingly (Non-Cochrane author).

Considering the capacity, skill and motivation of the author team in the selection of reviews to become LSRs was also emphasised.

LSRs require a large amount of author capacity and commitment which may limit feasibility. LSR teams should be from a well-known Cochrane group with high amount of LSR expertise (Cochrane managing editor).

The need for implementation policies supporting the roll-out of LSRs more broadly within Cochrane was also noted.

#### Publishing

Many of the challenges with current publishing processes raised by participants highlighted the need for better communication/promotion of LSRs.

Participants described the need to have a clear versioning system for updates, including those that do not have major changes. They highlighted the need to ensure all updates, irrespective of size, are clearly described within the review and suggested that a standardised way for readers to refer to previous updates is required.

Potentially using template text would result in less hands-on monthly support (Cochrane managing editor).

The way in which we describe the various updates in the review itself could be improved, as it is starting to get confusing to follow for the reader (Cochrane screening team member).

If new evidence is added but there is no change, should we still have the updated version online? Maybe different versions of the same DOI would work? The reader needs to be able to see different versions but in an easier / more user-friendly way. The latest version should appear first (rather than the first version) (Cochrane author).

When asked about opportunities for improvement/scale-up, ensuring living systematic reviews are both prominent and distinct from other reviews within the Cochrane Library was emphasised by many.

How to demonstrate what new things have been found and what makes it ‘living’. So identifying which studies are new, if included or not, and where. Or if they are waiting for updating. We need to clearly highlight what is new, what’s been found and what was included (Information specialist).

Participants also made suggestions for new publication options. Ideas included protocols for updates, and frequent, interim updates for the components of primary interest to policy makers and users, namely effect sizes and meta-analysis, with text changes happening later. This would reduce time to publish and produce ‘rapid access to key information slightly earlier’. The need for consistency between publishers about the publication process was also suggested:

It would be helpful to have consistency between publishers about the publication model and peer review process for LSRs. With F1000Research it’s easy given our existing model, but it’s not easy for many other publishers. Related to this, if a new team takes over an LSR, do they have to publish it with the same journal? Or can they go to a different one? If we are making use of meta-data and tools like Crossref properly, we could maybe do this. But it requires discussion and agreement across the publishing community (Non-Cochrane author).

#### Technology

In discussing opportunities for improvement and scale-up, many participants highlighted the need for better integration of technology within existing workflows. Further refinement of existing technologies and the development of new tools to manage the frequent searches, reduce workload and reduce human error were emphasised. Participants highlighted that further testing and refinement of citizen science and automation tools is also needed to ensure reliability. Specific opportunities for improvement included:

- Expanding citizen science opportunities (e.g. tasking Cochrane Crowd to screen other study designs; screen PICO statement eligibility criteria;
- Technologies/systems to auto-populate data (particularly the results tables) or conduct risk of bias assessment and data extraction; and
- Improving the existing technologies for search and screening to facilitate a more seamless workflow (e.g. facilitating the aggregation and de-duplication of search auto-alerts).

Many authors report they don’t have the resources, tech or tools to manage the frequent searches. We need solutions to offer authors (Non-Cochrane editorial team member).

Several participants suggested that maintaining the PRISMA flowchart could be improved. They suggested a living PRISMA flowchart that would allow the author team (and the reader) to see the progress of monthly searches in a live way. A suggested improvement to RevMan was to insert data in the analysis section and then have it all be linked and automatically updated.

#### Resources and support

When discussing the continuation and scale-up of LSRs, almost all participants indicated that feasibility is highly dependent on the addition/continuation of funding and resources and the provision of ongoing support. Participants highlighted the increased workload required of an LSR for the author and editorial teams (particularly copy-editing) and suggested that LSRs are not sustainable without additional funding.

Living systematic reviews are more costly than standard reviews in terms of resources needed. Additional funding and resources are needed to support long-term feasibility (Information specialist).

Participants emphasised the need to motivate author teams and to incentivise LSRs. Additional funding, addressing authorship challenges and providing access to ongoing support were identified as significant incentives to conducting an LSR and key to their feasibility and efficiency. In terms of support, participants discussed the need for the continuation of an LSR coordinator type role to oversee the process and provide assistance when required.

[We need someone to] facilitate communication within the implementation science community doing LSRs. Linking in with others doing LSRs making it explicit for groups going forward from here (Cochrane managing editor).

Other suggestions were for a team of experts supporting LSR groups or an LSR team to be built into each Cochrane Review Group to ‘update data and have clinical experts check it; something like the BMJ Clinical Evidence model where they send you all the evidence. This is really powerful, rapid fire turnaround, really useful, but requires resource’ (Cochrane coordinating editor).

#### Knowledge translation

Several participants expressed the need for the promotion of LSRs as an important factor to consider in terms of scale-up. They highlighted the need for promotion to both users of LSRs and funding groups.

How do we raise awareness and support uptake and use of LSRs? How do we get these reviews used? (Cochrane editor).

We need more education/awareness with funding groups and other important people about LSRs (Cochrane author).

We need to be able to demonstrate externally that a LSR is really changing and updating living guidelines and resulting in changing conclusions (Cochrane managing editor).

## Discussion and conclusions

Overall, there is considerable enthusiasm from contributors about the potential benefits and value of LSRs. Given the existence of sufficient support, on the basis of this evaluation, LSRs appear to be both an acceptable and feasible approach to keeping high-quality evidence synthesis continually up to date. The participants in this study described the importance of refining the methods and optimising the processes to support the feasibility of LSRs in the long term. Participants described their varying approaches to review production and highlighted the vital importance of an experienced, committed, enthusiastic team to manage the monthly requirements of an LSR. Participants spoke about the benefits of machine learning and citizen science approaches to manage the monthly workflow of citations and reduce time commitment, while also highlighting the shortcomings of these approaches and opportunities for improvement. They described the ongoing commitment required to conduct an LSR, the evolution of this process into a reliable, streamlined operation and the potential for LSRs to save time and effort.

There are challenges that need to be addressed for living systematic reviews to be sustainable and have maximum value. In light of their experiences, participants highlighted several factors to consider for the scale-up of living systematic reviews including:

- Clarifying roles, processes and expectations
- Providing resources and other incentives to increase motivation for undertaking and sustaining an LSR
- Better integration and awareness of technology to reduce human investment
- Having specific criteria about the appropriateness of a review to become an LSR
- Improving publication processes

The findings from this study will be used in discussions with the Cochrane community, key decision makers and people more broadly concerned with LSRs to identify and develop priorities for scale-up. Based on the success of the pilot reviews, Cochrane has committed to supporting LSRs in each of the Cochrane Networks. At last count, the Living Evidence Network (LEN) now consists of over 230 members, indicating the sustained and growing interest and commitment of people to the implementation of LSRs.

Limitations include the small number of reviews, and participants included in this study. The convergence of themes, the diverse roles of the respondents and the inclusion of studies (and participants) both within and outside of Cochrane however increases our confidence in the results. The interviewers were associated with running the LSR pilot evaluation study which may have influenced the results, however also increased the depth of the understanding. The limited duration of the evaluation period is also a limitation. It would be useful to have additional follow-up interviews as the LSR methods are processed and further refined.

Although several challenges remain, the feasibility and acceptability of LSR’s is clear. Funding to support those maintaining LSRs, streamlining and standardising publication processes and the development of further technologies to reduce workload are clear factors to consider for improvement and scale up.

## Data Availability

The full report on which this manuscript is based is available from https://community.cochrane.org/review-production/production-resources/living-systematic-reviews - pilots.

## Abbreviations

LEN: Living Evidence Network
LSR: Living systematic review
